# What is the most appropriate lipid profile ratio predictor for insulin resistance in each sex? A cross-sectional study in Korean populations (The Fifth Korea National Health and Nutrition Examination Survey)

**DOI:** 10.1186/s13098-015-0051-2

**Published:** 2015-06-28

**Authors:** A Ri Byun, Sang Wha Lee, Hong Soo Lee, Kyung Won Shim

**Affiliations:** Department of Family Medicine, Ewha Woman’s University Mokdong Hospital, 1071 AnYangCheon-Ro, YangCheon-Ku, Seoul South Korea

**Keywords:** LDL-cholesterol/HDL-cholesterol ratio, Non-HDL-cholesterol/HDL-cholesterol ratio, HOMA-IR, Insulin resistance, Blood cholesterol

## Abstract

**Background:**

Insulin resistance (IR) reduces reactivity of the target organ to blood insulin. Researchers have attempted to evaluate IR using various serum lipid concentration ratios. We aimed to determine the most strongly IR-predictive lipid profile ratios for each sex by studying associations between lipid concentration ratios and IR using data from the fifth Korea National Health and Nutrition Examination Survey (KNHANES V-1) 2010.

**Methods:**

Overall, 8958 individuals participated in health interview and examination surveys. Among them, 1910 individuals who completed physical examinations and 8-h fasting blood tests and were older than 20 years of age were enrolled (929 men and 981 women). The lipid-ratio-related study outcomes were the low-density lipoprotein cholesterol/high-density lipoprotein cholesterol (LDL-C/HDL-C), triglyceride (TG)/HDL-C, and non-HDL-C (LDL-C + TG/5)/HDL-C ratios. We divided subjects into 4 groups according to lipid profile ratio quartiles for a comparison of homeostasis model assessment (HOMA)-IR values. Regression analyses were performed after adjusting for the confounding factors of age, body mass index, and diabetes mellitus history.

**Results:**

HOMA-IR values tended to increase significantly along with LDL-C/HDL-C, TG/HDL-C, and non-HDL-C/HDL-C ratios in both sexes. In men, multiple linear regression analyses showed that after adjusting for confounding factors, a significant positive association remained only with the LDL-C/HDL-C ratio (*p* = 0.0238, R^2^ = 0.3605, root mean squared error [MSE] =0.3512). In women, multiple linear regression analyses showed that after adjusting for confounding factors, significant positive associations remained with the LDL-C/HDL-C (*p* < 0.0001, R-square = 0.2329, root MSE = 0.3776), TG/HDL-C (*p* = 0.0001, R^2^ = 0.2373, root MSE = 0.3766), and non-HDL-C/HDL-C ratios (*p* < 0.0001, R^2^ = 0.2456, root MSE = 0.3745).

**Conclusion:**

The LDL-C/HDL-C ratio in men and LDL-C/HDL-C, TG/HDL-C, and non-HDL-C/HDL-C ratios in women might be clinically significant predictors of IR in healthy Korean adults. However, additional large-scale studies are required to confirm these findings.

## Introduction

Insulin resistance (IR) is a condition in which the reactivity of a target organ to blood insulin is reduced, and is known to be the strongest predictor of type 2 diabetes mellitus occurrence [1]. IR is considered a core causal factor of hypertension, obesity, coronary artery disease, dyslipidemia, and metabolic syndrome [2] and is therefore actively studied worldwide.

IR can result from various genetic or acquired causes, including obesity, body fat distribution, reduced physical activity, malnutrition at birth, aging, pregnancy, drug use, chronic hyperglycemia, mitochondrial malfunction, inflammation, and stress [3–7]. In 1985, Matthews et al. first described the homeostasis model assessment for IR (HOMA-IR) as a useful and accurate method for IR quantification [8]. Since then, several clinicians have researched these data and found HOMA-IR to be a reliable marker of IR [9, 10].

Recently, researchers have actively attempted to evaluate IR using ratios of various serum lipid concentration levels, such as the total cholesterol/high-density lipoprotein cholesterol (HDL-C), low-density lipoprotein cholesterol (LDL-C)/HDL-C, and triglyceride (TG)/HDL-C ratios [11–14]. In addition, previous studies have suggested independent associations between IR and the LDL-C/HDL-C [13], TG/HDL-C, and total cholesterol (TC)/HDL-C ratios [11, 12, 14].

The serum concentrations of lipids differ according to sex [15, 16]. However, previous studies have evaluated data without distinguishing male and female subjects. We hypothesized that the strongest predictors of IR might differ between men and women. In the present study, we determined which lipid profile ratio served as the strongest IR predictive index for each sex by studying the associations between lipid profile ratios (e.g., LDL-C/HDL-C, TG/HDL-C, and non-HDL-C/HDL-C) and IR in Korean adults randomly selected from the Korea National Health and Nutrition Examination Survey (KNHANES) 2010.

## Methods

### Subjects and data collection

This study analyzed data from the fifth KNHANES (KNHANES V-1), a cross-sectional and nationally representative survey conducted by the Division of Chronic Disease Surveillance of the Korean Center for Disease Control and Prevention. KNHANES V-1 comprised 4 different surveys designed to evaluate the general health and nutrition status of the Korean population; these included a health interview survey, health behavior survey, health examination survey, and nutrition survey. During KNHANES V-1 2010, 8958 individuals participated in the health interview and health examination surveys. Among them, 1910 people older than 20 years of age who completed physical examinations and 8-h fasting blood tests were selected for the present study (929 men, 981 women). Subjects with missing data were excluded.

Health interviews and physical examinations were conducted at mobile exam centers, and nutrition surveys were conducted during visits to subjects’ households. Regarding drinking, the subjects were asked if they had not drunk any alcohol throughout their lives or if they had drunk at least 1 glass per month during the previous 1-year period. Regarding exercise, the subjects were asked to answer whether or not they had performed at least 3 sessions of very intense physical activity per week for 20 min or more per session or whether they had performed at least 5 sessions of moderate physical activity per week for 30 min or more per session.

### Physical measurements

Body mass index (BMI) was calculated by dividing the body weight (kg) by the height squared (m^2^). Waist circumference was measured during normal breathing at the center point between the lowest rib and pelvic iliac crest. Blood pressure was measured after the subjects had been seated for a 10-min rest period. Three systolic and diastolic blood pressure readings were recorded with a 5-min interval, and the average value was used for analysis.

### Anthropometric and laboratory parameter measurements

Plasma glucose and insulin levels after fasting for 8 h or more, as well as total cholesterol, TG, HDL-C, and LDL-C levels, were measured using a Hitachi Automatic Analyzer 7600 (Hitachi, Ltd., Tokyo, Japan). Glycated hemoglobin levels were measured using an HLC-723G7 auto analyzer (Tosoh Corporation, Minato, Japan). Blood samples were centrifuged, refrigerated at the examination site, and transferred in iceboxes to a central laboratory in Seoul on the day of collection.

### IR index

The IR index was calculated using the relatively simple and highly validated homeostasis model assessment (HOMA)-IR [8] as follows:

$$ \mathrm{HOMA}\hbox{-} \mathrm{I}\mathrm{R}=\mathrm{glucose}\ \left(\mathrm{mg}/\mathrm{dL}\right) \times \mathrm{insulin}\ \left(\mathrm{u}\mathrm{I}\mathrm{U}/\mathrm{mL}\right)/405 $$

### Statistical analysis

All analyses were performed separately for each sex. The KNHANES V-1 database sample used in this study was extracted via stratified, clustered, and systematic sampling. We considered strata, clusters, and weights in the statistical analysis. The SURVEYMEANS procedure was used to calculate averages and ratios, and the SURVEYREG and SURVEYFREQ procedures were used in the association analyses of continuous and categorical variables, respectively.

We used the LDL-C/HDL-C, TG/HDL-C, and non-HDL-C (LDL + TG/5)/HDL-C ratios as lipid concentration-ratio-related outcomes in the present study. We divided subjects into 4 groups according to lipid profile ratio quartiles to allow comparisons of HOMA-IR values. In the regression analysis, we adjusted for the confounding factors of age, BMI, waist circumference, and medical history of diabetes mellitus; Log (HOMA-IR) values were used because of the abnormal distribution of HOMA-IR values.

All statistical analyses were performed using SAS version 9.2 (SAS Institute, Cary, NC, USA), and significance was defined as a *p*-value < 0.05.

This study protocol conforms to the ethical guidelines of the 1975 Declaration of Helsinki as reflected in a priori approval by the Korea Centers for Disease Control and Prevention institutional review board in Korea (No. 2010-02CON-21-C).

## Results

### Baseline characteristics

The mean ages of the male and female subjects were 40.80 (95 % confidence interval (CI), 39.79–41.80) and 41.45 (95 % CI, 40.63–42.26) years, respectively. The mean LDL-C/HDL-C ratios were 2.40 (95 % CI, 2.33–2.46) for men and 2.04 (95 % CI, 1.99–2.09) for women. The mean TG/HDL-C ratios were 3.69 (95 % CI, 3.20–4.18) for men and 2.06 (95 % CI, 1.95–2.17) for women. The mean non-HDL-C/HDL-C ratios were 6.09 (95 % CI, 5.58–6.59) for men and 4.10 (95 % CI, 3.95–4.24) for women. The mean HOMA-IR values were 2.58 (95 % CI, 2.44–2.71) for men and 2.52 (95 % CI, 2.39–2.64) for women. There were significant differences between men and women in terms of smoking status, alcohol consumption, and physical activity. These results suggest that the men and women enrolled in this study experienced different lifestyles, with considerable influences on serum cholesterol levels [17, 18] (Table 1).

**Table 1:** Baseline characteristics

	Men				Women				*p*-value
	*N*	Mean	Lower C.I.	Upper C.I.	*N*	Mean	Lower C.I.	Upper C.I.	
Age (years)	929	40.80	39.79	41.80	981	41.45	40.63	42.26	0.2137
Height (cm)	929	171.50	170.93	172.07	981	158.40	157.95	158.85	<0.0001
Weight (kg)	929	71.30	70.38	72.22	981	57.75	57.04	58.47	<0.0001
BMI (kg/m^2^)	929	24.19	23.94	24.44	981	23.07	22.75	23.39	<0.0001
Waist Circumference (cm)	922	84.08	83.38	84.77	972	76.82	76.01	77.63	<0.0001
HDL-C (mg/dL)	929	49.90	48.91	50.89	981	56.77	55.85	57.70	<0.0001
LDL-C (mg/dL)	929	112.93	110.56	115.29	981	109.53	107.04	112.01	0.0518
Total Cholesterol (mg/dL)	929	187.02	184.09	189.95	981	184.53	181.56	187.50	0.2129
TG (mg/dL)	929	159.42	145.77	173.07	981	105.72	100.98	110.46	<0.0001
LDL-C/HDL-C Ratio	929	2.40	2.33	2.46	981	2.04	1.99	2.09	<0.0001
TG/HDL-C Ratio	929	3.69	3.20	4.18	981	2.06	1.95	2.17	<0.0001
Non-HDL-C (mg/dL)	929	272.35	258.69	286.00	981	215.25	209.32	221.17	<0.0001
Non-HDL-C/HDL-C Ratio	929	6.09	5.58	6.59	981	4.10	3.95	4.24	<0.0001
SBP (mmHg)	929	118.59	117.41	119.76	981	112.61	111.49	113.74	<0.0001
DBP (mmHg)	929	77.65	76.65	78.66	981	71.87	71.11	72.64	<0.0001
Fasting Glucose (mg/dL)	929	98.67	96.33	101.01	981	93.31	92.11	94.52	<0.0001
Fasting Insulin (uIU/mL)	929	10.46	10.00	10.91	981	10.69	10.32	11.06	0.4121
HOMA-IR	929	2.58	2.44	2.71	981	2.52	2.39	2.64	0.5048
	N	%	Lower C.I.	Upper C.I.	N	%	Lower C.I.	Upper C.I.	
Hypertension									0.3511
No	729	84.48	82.10	86.85	796	85.92	83.96	87.88	
Yes	190	15.52	13.15	17.90	180	14.08	12.12	16.04	
Hyperlipidemia									0.9149
No	833	92.51	90.47	94.55	867	92.63	90.97	94.30	
Yes	85	7.49	5.45	9.53	109	7.37	5.70	9.03	
Diabetes									0.1617
No	842	93.53	91.79	95.27	920	95.22	93.69	96.75	
Yes	76	6.47	4.73	8.21	56	4.78	3.25	6.31	
Smoking									<0.0001
No	496	50.66	46.71	54.62	928	94.58	92.81	96.35	
Yes	426	49.34	45.38	53.30	50	5.42	3.65	7.19	
Alcohol Use									<0.0001
No	192	20.59	17.48	23.69	587	55.83	51.53	60.12	
Yes	722	79.41	76.31	82.52	384	44.17	39.88	48.47	
Exercise: high									<0.0001
No	730	77.47	74.01	80.93	855	87.11	84.29	89.94	
Yes	191	22.53	19.07	26.00	121	12.89	10.06	15.71	
Exercise: mid									0.0798
No	817	87.19	84.46	89.93	876	90.01	87.86	92.17	
Yes	105	12.81	10.07	15.54	100	9.99	7.83	12.14	

*p-*values were calculated using the SURVEYMEANS (for continuous values) and SURVEYFREQ procedures (for categorical values)

*BMI* Body mass index, *LDL-C* Low-density lipoprotein cholesterol, *HDL-C* High-density lipoprotein cholesterol, *SBP* Systolic blood pressure, *DBP* Diastolic blood pressure, *HOMA-IR* Homeostasis model assessment of insulin resistance, *C.I.* Confidence interval

### Mean comparison of HOMA-IR values among LDL-C/HDL-C, TG/HDL-C, and non-HDL-C/HDL-C ratio quartile groups

We divided the subjects into sets of 4 quartile groups according to the LDL-C/HDL-C ratio (first, second, third, and fourth quartile groups:<1.64000, 1.64000–2.15895, 2.15895–2.78788, and ≥ 2.78788, respectively), TG/HDL-C ratio (first, second, third, and fourth quartile groups:<1.25581, 1.25581–2.04302, 2.04302–3.50000, and ≥ 3.50000, respectively), and non-HDL-C/HDL-C ratio (first, second, third, and fourth quartile groups:<3.07042, 3.07042–4.28846, 4.28846–6.31818, and ≥ 6.31818, respectively) (Table 2).

**Table 2:** Mean comparison of HOMA-IR values among LDL-C/HDL-C, TG/HDL-C, and non-HDL-C/HDL-C ratio quartile groups

(A) Men	1st Quartile			2nd Quartile			3rd Quartile			4th Quartile			*p*-value
LDL-C/HDL-C^a^	*N* = 183			*N* = 210			*N* = 251			*N* = 285			
	Mean	Lower C.I	Upper C.I	Mean	Lower C.I	Upper C.I	Mean	Lower C.I	Upper C.I	Mean	Lower C.I	Upper C.I	
HOMA-IR	2.23	2.09	2.37	2.51	2.08	2.94	2.54	2.34	2.74	2.90	2.67	3.14	<0.0001
Log (HOMA-IR)	0.72	0.66	0.79	0.78	0.69	0.87	0.83	0.77	0.90	0.95	0.89	1.02	<0.0001
TG/HDL-C^b^	*N* = 137			*N* = 200			*N* = 257			*N* = 335			
	Mean	Lower C.I	Upper C.I	Mean	Lower C.I	Upper C.I	Mean	Lower C.I	Upper C.I	Mean	Lower C.I	Upper C.I	
HOMA-IR	1.99	1.80	2.17	2.29	2.10	2.48	2.46	2.26	2.67	3.13	2.83	3.43	<0.0001
Log (HOMA-IR)	0.63	0.56	0.70	0.74	0.68	0.81	0.80	0.73	0.86	1.02	0.97	1.08	<0.0001
Non-HDL-C/HDL-C^c^	*N* = 146			*N* = 204			*N* = 241			*N* = 338			
	Mean	Lower C.I	Upper C.I	Mean	Lower C.I	Upper C.I	Mean	Lower C.I	Upper C.I	Mean	Lower C.I	Upper C.I	
HOMA-IR	1.99	1.82	2.17	2.23	2.07	2.40	2.59	2.38	2.81	3.08	2.77	3.40	<0.0001
Log (HOMA-IR)	0.62	0.55	0.69	0.73	0.67	0.79	0.85	0.78	0.91	1.00	0.94	1.07	<0.0001
(B) Women	1st Quartile			2nd Quartile			3rd Quartile			4th Quartile			*p*-value
LDL-C/HDL-C^d^	*N* = 294			*N* = 268			*N* = 228			*N* = 191			
	Mean	Lower C.I	Upper C.I	Mean	Lower C.I	Upper C.I	Mean	Lower C.I	Upper C.I	Mean	Lower C.I	Upper C.I	
HOMA-IR	2.14	2.00	2.28	2.37	2.21	2.54	2.56	2.38	2.74	3.44	2.90	3.97	<0.0001
Log (HOMA-IR)	0.69	0.63	0.75	0.78	0.72	0.84	0.86	0.79	0.92	1.06	0.97	1.15	<0.0001
TG/HDL-C^e^	*N* = 341			*N* = 277			*N* = 220			*N* = 143			
	Mean	Lower C.I	Upper C.I	Mean	Lower C.I	Upper C.I	Mean	Lower C.I	Upper C.I	Mean	Lower C.I	Upper C.I	
HOMA-IR	2.23	2.03	2.44	2.28	2.16	2.41	2.64	2.45	2.83	3.61	3.10	4.13	<0.0001
Log (HOMA-IR)	0.70	0.65	0.76	0.75	0.70	0.81	0.87	0.80	0.94	1.16	1.07	1.25	<0.0001
non-HDL-C/HDL-C^f^	*N* = 332			*N* = 275			*N* = 233			*N* = 141			
	Mean	Lower C.I	Upper C.I	Mean	Lower C.I	Upper C.I	Mean	Lower C.I	Upper C.I	Mean	Lower C.I	Upper C.I	
HOMA-IR	2.15	2.00	2.30	2.29	2.17	2.41	2.77	2.44	3.09	3.64	3.10	4.18	<0.0001
Log (HOMA-IR)	0.69	0.63	0.75	0.76	0.71	0.81	0.89	0.82	0.96	1.16	1.06	1.26	<0.0001

*p*-values were obtained via the SURVEYMEANS procedure

*LDL-C* Low-density lipoprotein cholesterol, *HDL-C* High-density lipoprotein cholesterol, *TG* Triglyceride, *HOMA-IR* Homeostasis model assessment of insulin resistance, *C.I.* Confidence interval

^a^1st quartile group (<1.64000), 2nd quartile group (1.64000–2.15895), 3rd quartile group (2.15895–2.78788), 4th quartile group (≥2.78788)

^b^1st quartile group (<1.25581), 2nd quartile group (1.25581–2.04302), 3rd quartile group (2.04302–3.50000), 4th quartile group (≥3.50000)

^c^1st quartile group (<174), 2nd quartile group (174–223), 3rd quartile group (223–291), 4th quartile group (≥291)

^d^1st quartile group (<1.64000), 2nd quartile group (1.64000–2.15895), 3rd quartile group (2.15895–2.78788), 4th quartile group (≥2.78788)

^e^1st quartile group (<1.25581), 2nd quartile group (1.25581–2.04302), 3rd quartile group (2.04302–3.50000), 4th quartile group (≥3.50000)

^f^1st quartile group (<3.07042), 2nd quartile group (3.07042–4.28846), 3rd quartile group (4.28846–6.31818), 4th quartile group (≥6.31818)

Statistically significant differences were observed within each sex among all 3 sets of quartile groups (mean LDL-C/HDL-C, TG/HDL-C, and non-HDL-C/HDL-C ratios; *p*-values were obtained via the SURVEYMEANS procedure). In both sexes, HOMA-IR values tended to increase as the LDL-C/HDL-C, TG/HDL-C, and non-HDL-C/HDL-C ratios increased.

### Multiple linear regression model of log transformed HOMA-IR values in men

Table 3 lists the results of analytical evaluations of the associations of individual lipid measure ratios (LDL-C/HDL-C ratio, TG/HDL-C ratio and non-HDL-C/HDL-C ratio) with HOMA-IR according to a multiple linear regression test. In men, multiple linear regression analyses revealed that after adjusting for the confounding factors of age, BMI, and medical history of diabetes mellitus, a significant positive association was only observed with the LDL-C/HDL-C ratio (*p*-value = 0.0238, adjusted R^2^ = 0.3588, root mean square error [MSE] =0.3512).

**Table 3:** Results of the multiple linear regression model for log transformed HOMA-IR

	Men					Women				
	Estimate	Standard error	*p*-value	Adjusted R^2^	Root MSE	Estimate	Standard error	*p*-value	Adjusted R^2^	Root MSE
*Model with LDL-C/HDL-C*				0.3588	0.3512				0.2309	0.3776
Age	−0.0010	0.0012	0.3953			−0.0027	0.0013	0.0451		
BMI	0.0429	0.0086	<0.0001			0.0247	0.0100	0.0149		
Waist Circumference	0.0101	0.0028	0.0007			0.0066	0.0039	0.0919		
Diabetes^a^	0.3911	0.0621	<0.0001			0.3578	0.1014	0.0005		
LDL-C/HDL-C Ratio	0.0383	0.0168	0.0238			0.1015	0.0242	<0.0001		
*Model with TG/HDL-C*				0.3641	0.3497				0.2353	0.3766
Age	−0.0010	0.0013	0.4126			−0.0025	0.0013	0.0529		
BMI	0.0445	0.0083	<0.0001			0.0256	0.0101	0.010		
Waist Circumference	0.0097	0.0029	0.0009			0.0065	0.0042	0.1206		
Diabetes^a^	0.3868	0.0635	<0.0001			0.3415	0.0998	0.0008		
TG/HDL-C Ratio	0.0086	0.0070	0.2222			0.0485	0.0124	0.0001		
*Model with non-HDL-C/HDL-C*				0.3662	0.3491				0.2437	0.3745
Age	−0.0011	0.0013	0.3690			−0.0030	0.0013	0.0224		
BMI	0.0439	0.0084	<0.0001			0.0242	0.0100	0.0164		
Waist Circumference	0.0096	0.0029	0.0009			0.0060	0.0041	0.1421		
Diabetes^a^	0.3898	0.0632	<0.0001			0.3394	0.0969	0.0006		
Non-HDL-C/HDL-C Ratio	0.0091	0.0069	0.1907			0.0448	0.0095	<0.0001		

*p-*values were evaluated using the SURVEYMEANS procedure (LDL-C/HDL-C, TG/HDL-C, and non-HDL-C/HDL-C ratios were treated as continuous values)

*R* ^*2*^ Root-square, *Root MSE* Root mean squared error, *HOMA-IR* Homeostasis model assessment of insulin resistance, *LDL-C/HDL-C* Low-density lipoprotein cholesterol/high-density lipoprotein cholesterol ratio, *TG/HDL-C* Triglyceride/high-density lipoprotein cholesterol ratio, *non-HDL-C/HDL-C* Non-high-density lipoprotein cholesterol (LDL-C + triglyceride)/HDL-C ratio, *BMI* Body mass index

^a^Diabetes was defined as a medical history of diabetes mellitus

### Multiple linear regression model of log transformed HOMA-IR values in women

In women, multiple linear regression analyses showed that after adjusting for the confounding factors of age, BMI, and medical history of diabetes mellitus, significant positive associations were observed with the LDL-C/HDL-C ratio (*p*-value <0.0001, adjusted R^2^ = 0.2309, root MSE = 0.3776), TG/HDL-C ratio (*p*-value = 0.0001, adjusted R^2^ = 0.2353, root MSE = 0.3766), and non-HDL-C/HDL-C ratio (*p*-value < 0.0001, adjusted R^2^ = 0.2437, root MSE = 0.3745) (Table 3).

## Discussion

This study was performed to evaluate sex-based associations between lipid concentration ratios (e.g., LDL-C/HDL-C, TG/HDL-C, and non-HDL-C/HDL-C) and HOMA-IR values in order to determine the strongest predictor of IR in each sex. According to the results, only the LDL-C/HDL-C ratio was significantly and positively associated with HOMA-IR values in men. On the other hand, the LDL-C/HDL-C (*p*-value < 0.0001, adjusted R^2^ = 0.2309, root MSE = 0.3776), TG/HDL-C (*p*-value = 0.0001, adjusted R^2^ = 0.2353, root MSE = 0.3766), and non-HDL-C/HDL-C ratios (*p*-value < 0.0001, adjusted R^2^ = 0.2437, root MSE = 0.3745) were all significantly and positively associated with HOMA-IR values in women.

In most cases, patients with IR have hypertriglyceridemia and low HDL-C levels because insulin affects the metabolism of very low-density lipoprotein cholesterol (VLDL-C) and HDL-C [19]. Systemically circulating insulin inhibits the secretion of VLDL-C and stimulates the activation of lipoprotein lipase (LPL). However, if IR occurs, more TG is synthesized in the liver, thus increasing apolipoprotein B levels via increased fat generation, increasing VLDL-C secretion, and decreasing the concentration and activity of LPL in peripheral tissues, especially adipose tissue. Ultimately, the decrease in HDL-C levels occurs as a secondary process following an increase in TG [20].

Many previous studies have suggested various lipid ratios as simple and useful parameters for determining IR. For example, when TG levels and TG/HDL-C ratios were compared and analyzed in 50 Caucasian Americans, the areas under the receiver-operating characteristic curves (AUCs) were 0.763 and 0.770, respectively, suggesting that the TG/HDL-C ratio was a significant predictor of IR. However, the significance of this ratio was low among African-Americans [11]. In another study in which the TG/HDL-C and total cholesterol/HDL-C ratios were compared in 2,014 patients, the TG/HDL-C ratio was reported to be an imperfect but statistically significant (AUC = 0.745, *p*-value < 0.001) predictor of the risks of IR and coronary artery disease [12]. According to a recent analysis of 1,393 Japanese subjects, the LDL-C/HDL-C ratio was found to be a significant predictor of IR among non-obese subjects (AUC = 0.74, *p*-value < 0.001), with a cut-off value of 2.14 [13]. Therefore, the best lipid profile ratio for predicting IR remains controversial.

We focused on sex-based differences to determine the most IR-predictive lipid profile ratios. One previous study suggested that women and men have significantly different lipid, apoprotein, and lipoprotein values (lower BMI, TG, and total cholesterol/HDL-C ratio values and higher HDL-C values in women vs. men) [21]. Another study also suggested that women have higher apolipoproteinA-I production rates, compared with men [22]. The results of the present study support these previously reported differences (Table 3). In the present study, women had significantly lower BMI and TG values and significantly higher HDL-C values. Therefore, we performed a multiple regression analysis of each lipid profile ratio in each sex.

The present study has the following strengths. In this study, the best predictor of IR was examined in each sex, an investigation that has not been previously reported. Accordingly, only the LDL-C/HDL-C ratio was found to be significantly associated with HOMA-IR values in men, whereas a regression model revealed that the LDL-C/HDL-C, TG/HDL-C, non-HDL-C/HDL-C ratios were clinically significant in women. In addition, as only 6.91 % (132) of the subjects had a medical history of diabetes mellitus, the results of the present study can be fully applied to healthy adult Korean groups in clinical settings.

The limitations of this study are as follows. First, as this was a cross-sectional study based on data from KNHANES 2010, inaccurate information and recall errors might have been introduced through the interview methods and self-administered questionnaires. Second, as other risk factors of IR such as family history, medical history of gestational diabetes mellitus, or presence of an inflammatory reaction were not considered, these might have affected the outcomes. Finally, cut-off values for the lipid profile ratios found to be optimum predictors of IR could not be determined in this study; this issue will require further evaluation in an additional large-scale study in the future.

## Conclusions

We suggest that the LDL-C/HDL-C ratio and the LDL-C/HDL-C, TG/HDL-C, and non-HDL-C/HDL-C ratios could be clinically significant predictors of IR in healthy adult men and women, respectively. However, additional large-scale studies will be required to evaluate the optimum cut-off values for the lipid profile ratios used to determine IR in each sex.
